# The impact of physical exercise on adolescents’ mobile phone dependency: the serial mediating role of self-esteem and depression

**DOI:** 10.3389/fpsyg.2025.1471657

**Published:** 2025-02-10

**Authors:** Jingtao Wu, Yanhong Shao, Wanli Zang

**Affiliations:** ^1^School of Physical Education, Leshan Normal University, Leshan, China; ^2^Xiangshui Teacher Development Center, Yancheng, China; ^3^Postgraduate School, Harbin Sport University, Harbin, China

**Keywords:** physical exercise, mobile phone dependency, self-esteem, depression, middle school students

## Abstract

**Objective:**

The objective of this study is to examine the impact of physical exercise on the prevalence of mobile phone dependency among middle school students, as well as to delineate the serial mediating roles of self-esteem and depression within this relationship.

**Methods:**

A convenient cluster random sampling method was employed to conduct a questionnaire survey among 3,786 middle school students from Guangdong, Sichuan, Zhejiang, Henan, and other provinces. Measurements were taken using the Physical Activity Rating Scale (PARS-3), the Self-esteem Scale, the Depression Scale, and the Mobile Phone Dependency Scale.

**Results:**

Physical exercise was significantly negatively correlated with mobile phone dependency (*r* = −0.400, *p* < 0.001) and depression (*r* = −0.400, *p* < 0.001), and positively correlated with self-esteem (*r* = 0.257, *p* < 0.001). Mobile phone dependency was significantly positively correlated with depression (*r* = 0.540, *p* < 0.001) and negatively correlated with self-esteem (*r* = −0.129, *p* < 0.001). Depression was negatively correlated with self-esteem (*r* = −0.396, *p* < 0.001). Mediation analysis revealed that self-esteem and depression significantly mediated the relationship between physical exercise and mobile phone dependency, with a total indirect effect of −0.116 (95% *CI* = [−0.239, −0.140]), accounting for 100% of the total effect. Specifically, the indirect effect through “physical exercise influencing depression, which then affects mobile phone dependency” was −0.076 (95% *CI* = [−0.224, −0.128]), accounting for 65.52% of the total effect; the indirect effect through “physical exercise influencing self-esteem, which then influences depression, and in turn affects mobile phone dependency” was −0.040 (95% *CI* = [−0.063, −0.027]), accounting for 34.48%; the indirect effect through “physical exercise influencing self-esteem, which then affects mobile phone dependency” was not significant (95% CI included 0).

**Conclusion:**

Physical exercise can directly reduce the dependency of middle school students on mobile phones, and it can also indirectly reduce mobile phone dependency by enhancing self-esteem and reducing levels of depression.

## 1 Introduction

With the advent of the information age, the use of smartphones has become widespread, and many individuals are obsessed with mobile games and virtual social spaces, exhibiting behaviors of mobile phone dependency (Salehan and Negahban, 2013). Mobile phone dependency has become an increasingly severe problem among adolescents (Wu et al., 2024). Mobile phone dependency refers to an individual’s long-term use of mobile phones and an inability to control their mobile phone usage time (Zhang et al., 2020). Prolonged dependency on mobile phones not only affects adolescents’ academic performance (Yadav et al., 2021), but also leads to physiological disorders, interpersonal relationship issues, and difficulties in social adaptation (Rashid et al., 2021; Chun, 2013; Wang et al., 2021). Therefore, studying adolescents’ mobile phone dependency has certain theoretical and practical significance.

In recent years, physical exercise has emerged as a proactive lifestyle choice, garnering increasing attention for its positive effects on the physical and mental health of the general public (Palenzuela-Luis et al., 2023). And it has also been recognized as an important external factor in adolescents’ mobile phone dependency (Xu, 2023). Physical exercise refers to a series of purposeful, planned, and repetitive physical activities (Zhu, 2017). Studies have indicated that the more frequent the physical exercise, the less adolescents are dependent on mobile phones (Xu and Tang, 2024).

Although researchers have proposed that these three factors influence adolescents’ mobile phone dependency behaviors, the mechanism by which physical exercise affects mobile phone dependency through self-esteem and depression has not yet been explored. Therefore, this study aims to investigate the relationship between physical exercise, self-esteem, depression, and mobile phone dependency, as well as the mediating role of self-esteem and depression in the relationship between physical exercise and mobile phone dependency. The originality of this study lies in exploring the specific mechanism by which physical exercise affects adolescents’ mobile phone dependency through self-esteem and depression, filling a gap in existing research. The significance of this research lies in its potential to contribute to the understanding of the complex interplay between physical exercise, psychological well-being, and mobile phone dependency among adolescents. By elucidating the mediating roles of self-esteem and depression, this study can offer insights into the development of targeted interventions that may mitigate the negative impacts of excessive mobile phone use.

### 1.1 The relationship between physical exercise and mobile phone dependency

There is a correlation between physical exercise and mobile phone dependency. The Self-Determination Theory (Ryan and Deci, 2008) posits that fulfilling the psychological needs of autonomy, competence, and relatedness is crucial for intrinsic motivation and well-being. Kwon and Jin (2019) suggest that physical exercise can meet these needs, promoting mental health and prosocial behavior. Vega-Díaz and González-García (2024) note that leisure-time exercise can enhance autonomy, pleasure, and competence, while also fostering social interaction and relatedness. Conversely, Zhao et al. (2023) argue that excessive mobile phone use is a maladaptive strategy for fulfilling these needs, leading to social isolation and dependency (Cheng et al., 2020). Xu and Tang (2024) propose that regular exercise could reduce phone dependency by enhancing self-control and efficacy. Peng (2024) finds that achieving these psychological needs in real social contexts significantly reduces phone dependency. Gong et al. (2023) review the literature, indicating that both physical exercise and phone dependency are influenced by various factors, with Zhu et al. (2023) suggesting that exercise levels could differentially affect phone dependency. This study hypothesizes that regular exercise, by satisfying basic psychological needs, can mitigate mobile phone dependency symptoms.

*H1*: Physical exercise can negatively predict the mobile phone dependency among middle school students.

### 1.2 The mediating role of self-esteem between physical exercise and mobile phone dependency

The mediating role of self-esteem in the relationship between physical exercise and mobile phone dependency is a key focus within the Self-Determination Theory framework (Shi et al., 2021). Self-esteem, as a central psychological construct, significantly impacts psychological health and social behavior (Mun and Lee, 2023). Research indicates a positive correlation between self-esteem and physical exercise (Fernandes et al., 2024), with exercise fulfilling psychological needs and enhancing self-esteem (Dijkslag et al., 2024). Conversely, a negative correlation exists between self-esteem and mobile phone dependency, with low self-esteem individuals more likely to seek gratification and social interaction through phones (Li, 2023; Kong et al., 2022). This behavior, however, fails to genuinely boost satisfaction or self-esteem, instead deepening phone dependency (Kong et al., 2022).

Given this, self-esteem is inferred to mediate the impact of physical exercise on mobile phone dependency, with exercise indirectly reducing dependency by bolstering self-esteem. Regular physical exercise is assumed to increase self-esteem and self-efficacy, reducing phone usage and enabling individuals to confront life’s challenges with more courage (Liu et al., 2022). A longitudinal study by Huo (2021) supports this, showing that exercise reduces phone dependency in college students by enhancing self-esteem. This empirical evidence supports the mediating effect of self-esteem. Thus, this study hypothesizes:

*H2*: Self-esteem mediates the influence of physical exercise on mobile phone dependency among middle school students.

### 1.3 The mediating role of depression between physical exercise and mobile phone dependency

Depression is recognized as a significant mediator in the relationship between physical exercise and mobile phone dependency, with a substantial body of research highlighting its role in internalizing problem behaviors (Du et al., 2024). Depression, a mood disorder impacting quality of life, is negatively correlated with physical exercise, which can alleviate depressive symptoms through physiological, psychological, and social mechanisms (Elgendy et al., 2024; Chen et al., 2022; Pangkahila et al., 2016; Xu and Tang, 2024; Hou et al., 2024; Runsen et al., 2020). Conversely, depression is positively correlated with mobile phone dependency, with hypotheses suggesting that individuals may use phones to escape real-life troubles, vent negative emotions, or seek social interaction, leading to addictive behaviors (Park and Yoo, 2023; Lee et al., 2023; Jiang et al., 2023).

Depression is inferred to mediate the impact of physical exercise on mobile phone dependency, with exercise potentially reducing dependency by lessening depressive symptoms. Consistent physical activity is believed to fulfill psychological and physiological needs, reduce depressive emotions, and decrease reliance on phones for emotional regulation and social compensation. Empirical evidence supports this, with studies showing a negative correlation between college students’ phone dependency and exercise, mediated by depression (Liu et al., 2022), and a longitudinal study indicating that regular exercise alleviates depressive emotions and reduces phone use (Zhong et al., 2021). Thus, this research hypothesizes:

*H3*: Depression mediates the impact of physical exercise on mobile phone dependency among middle school students.

### 1.4 The serial mediating role of self-esteem and depression in the impact of physical exercise on mobile phone dependency

Scholarly research, including Romero-Rodríguez et al. (2020), highlights self-esteem and depression as key mediators in the link between physical exercise and mobile phone dependency. Evidence shows a strong connection between these two factors, influencing mobile phone use behaviors (Jose et al., 2024). According to the protective-risk factor model by Gutiérrez-Cobo et al. (2018), self-esteem acts as a protective factor that helps mitigate the impact of risk factors like depression on psychological well-being. High self-esteem aids in adapting to the environment and maintaining well-being, while depression is a risk factor. When depressive symptoms appear, stronger self-esteem can reduce negative emotions and the likelihood of developing mobile phone dependency.

Research confirms that self-esteem is a significant predictor of negative emotions and depression (Gilbert et al., 2024). Studies, including Jüllig et al. (2024), show that higher self-esteem is associated with increased self-efficacy and a lower risk of depressive moods. Lei et al. (2024) find that those with lower self-esteem are more prone to negative cognition and self-evaluation, increasing the risk of rumination and depression. In contrast, individuals with higher self-esteem use positive coping strategies to reduce the impact of depressive symptoms.

Moreover, empirical research, including Liu J. et al. (2024), Liu X. et al. (2024) and Gathier et al. (2024), confirms a strong link between depression and mobile phone dependency. Depressed individuals often turn to mobile phones for comfort, increasing their dependency. The dual-system model explained by Wojciechowski and Krupa (2024) suggests that depression enhances the impulsive system, driving the pursuit of immediate gratification and reducing the cognitive control to resist mobile phone use, leading to weakened self-control.

Physical exercise, a proactive lifestyle choice, impacts self-esteem and depression through various mechanisms. Wang et al. (2024) note that regular activity improves body self-concept and self-confidence, raising self-esteem. Tsartsapakis et al. (2024) find that exercise also improves emotional states and moods under stress, reducing depression risk. Dadiotis and Roussos (2024) suggest that exercise can indirectly boost self-esteem by increasing self-efficacy and decreasing negative emotions and depression.

**Figure 1:**
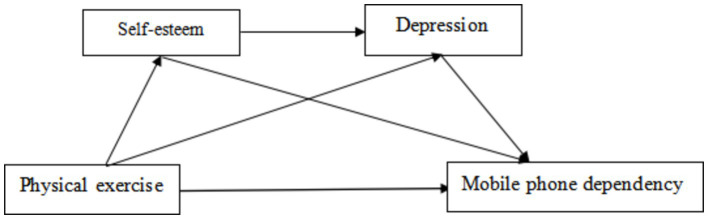
Research hypothesis model diagram.

Despite established links between physical exercise, self-esteem, depression, and mobile phone dependency, the internal dynamics and mechanisms require further exploration (Figure 1). This study aims to investigate these relationships, with physical exercise as the independent variable, mobile phone dependency as the dependent variable, and self-esteem and depression as mediators, to uncover the mechanisms linking exercise and mobile phone dependency. Consequently, this study posits the following research hypothesis:

*H4*: Self-esteem and depression play a serial mediating role in the impact of physical exercise on mobile phone dependency among middle school students.

## 2 Research methodology

### 2.1 Participants and procedures

This study employed a convenient cluster random sampling method to survey junior and senior high school students in Guangdong, Sichuan, Zhejiang, Henan, and other provinces from May to June 2024. To ensure the participants’ right to be informed and to adhere to the voluntary principle of their participation in the survey, confidentiality measures regarding the survey content were communicated before the assessment, emphasizing the use of the data collected. During the survey, the presence of the class teacher was secured to assist in organizing the survey and to remind students not to omit any relevant information. Questionnaires were distributed on-site and collected after 10 min of completion. A total of 3,786 questionnaires were collected, and based on the principles of completeness, regularity of response, and absence of omissions, each questionnaire was scrutinized. After excluding invalid questionnaires, 3,426 valid questionnaires were retrieved, yielding a valid recovery rate of 90.49%. Among them, there were 1,781 males (52.0%) and 1,645 females (48.0%); 1,856 junior high school students (54.2%) and 1,570 senior high school students (45.8%); 1,976 from urban areas (57.7%) and 1,450 from rural areas (42.3%). This survey was approved by the Ethics Committee of Leshan Normal College before implementation, with the approval number 202405017.

### 2.2 Research instruments

#### 2.2.1 Physical activity rating scale-3

This study used the Physical Activity Level Assessment Scale revised by domestic scholars Liang (1994) to measure the level of physical exercise among middle school students. The scale includes three dimensions: exercise intensity, duration, and frequency of participation, each consisting of one item. The scale uses a 5-point Likert scoring system, with the scoring range for exercise intensity and participation frequency being 1–5 points, and for duration 0–4 points. The level of physical exercise is obtained by multiplying the scores of these three dimensions, with higher scores indicating a higher level of physical exercise. In this study, the internal consistency coefficient (Cronbach’s α) was 0.78, indicating high internal consistency.

#### 2.2.2 Mobile phone dependency scale

This study used the Mobile Phone Dependency Scale (MPDAS) for middle school students developed by Tao et al. (2013) to measure the degree of mobile phone dependency. The scale includes 16 items divided into three dimensions: Compulsive Use (6 items), Withdrawal Symptoms (5 items), and Functional Impairment (5 items). The scale uses a 5-point Likert scoring system, ranging from “1 = Not at all” to “5 = Completely.” It includes 3 reverse-scored items (questions 4, 9, and 15) that require reverse scoring. The total score ranges from 16 to 80, with higher scores indicating more severe mobile phone dependency. The score range for each dimension is as follows: Compulsive Use 6–30 points, Withdrawal Symptoms 5–25 points, and Functional Impairment 5–25 points. A score above the 75th percentile of the scale is considered to indicate significant mobile phone dependency symptoms. In this study, the scale’s Cronbach’s α coefficient was 0.91, indicating high internal consistency. Confirmatory factor analysis results showed: CMIN/DF (Chi-square/degrees of freedom) = 1.95, GFI (Goodness-of-Fit Index) = 0.92, AGFI (Adjusted Goodness-of-Fit Index) = 0.90, CFI (Comparative Fit Index) = 0.95, RMSEA (Root Mean Square Error of Approximation) = 0.043, indicating good structural validity.

#### 2.2.3 Self-esteem scale

This study used the Chinese version of the Rosenberg Self-Esteem Scale revised by Li (2004) to assess the self-esteem level of middle school students. The scale includes 10 items, with 5 positive statements and 5 negative statements, using a 4-point Likert scoring method (1 = Strongly Disagree, 4 = Strongly Agree). The total score ranges from 10 to 40, with higher scores indicating higher self-esteem. The scale showed good reliability and validity in this study, with a Cronbach’s α coefficient of 0.89. Confirmatory factor analysis indicated good structural validity: CMIN/DF (Chi-square/degrees of freedom) = 2.13, GFI (Goodness-of-Fit Index) = 0.94, AGFI (Adjusted Goodness-of-Fit Index) = 0.91, CFI (Comparative Fit Index) = 0.96, RMSEA (Root Mean Square Error of Approximation) = 0.048.

#### 2.2.4 Depression scale

This study used the Depression subscale of Depression-Anxiety-Stress Scales (DASS-21) revised by Gong et al. (2010), to assess depressive symptoms among middle school students. The Depression subscale includes 7 items, using a four-point scoring method (0 = Not at all, 3 = Completely). The total score ranges from 0 to 21, with higher scores indicating more severe depressive symptoms. In this study, the depression subscale showed excellent internal consistency, with a Cronbach’s α coefficient of 0.87. Confirmatory factor analysis indicated good structural validity: CMIN/DF (Chi-square/degrees of freedom) = 1.82, GFI (Goodness-of-Fit Index) = 0.95, AGFI (Adjusted Goodness-of-Fit Index) = 0.92, CFI (Comparative Fit Index) = 0.97, RMSEA (Root Mean Square Error of Approximation) = 0.037. These indicators all suggest that the Depression subscale has good reliability and validity in this study and is suitable for assessing depressive symptoms in middle school student populations.

### 2.3 Data processing

The cleaned data were imported into the SPSS 26.0 statistical analysis software. Descriptive statistical analysis was conducted initially to calculate the means and standard deviations of each variable. Pearson correlation analysis was utilized to test the correlation between variables. Tests for homogeneity of variance and multicollinearity diagnostics were performed, and the variance inflation factor (VIF) was checked to determine the presence of multicollinearity issues. Multiple linear regression analysis was conducted with physical exercise as the independent variable, mobile phone dependency as the dependent variable, and self-esteem and depression as mediating variables. The Process macro (version 4.1) plugin with Bootstrap method was employed for serial mediation analysis (Li and Beretvas, 2013), with Model 6 specified as follows; X = physical exercise, M1 = self-esteem, M2 = depression, Y = mobile phone dependency, and Bootstrap Samples were drawn 5,000 times. Both direct and indirect effects were analyzed to assess the serial mediating role of self-esteem and depression in the process by which physical exercise affects mobile phone dependency.

## 3 Results and analysis

### 3.1 Common method bias test

To assess the impact of common method bias in this study, a common method bias test was conducted. Initially, reverse-scored items and outliers in the questionnaire were transposed and eliminated. Subsequently, a Harman single-factor test was utilized to examine common method bias (Podsakoff et al., 2003). All variables were subjected to an exploratory factor analysis (EFA) using the principal component analysis method. The examination of the unrotated factors revealed that the first unrotated principal component accounted for 28.63% of the variance, which is below the critical threshold of 40%. This indicates that common method bias is not a serious concern in this study.

### 3.2 Basic characteristics of demographic variables

As shown in Table 1, discernible variations are noted across gender, age, and grade level in relation to the observed variables. Notably, significant gender-based disparities are evident in the realms of physical exercise and mobile phone dependency (*p* < 0.001), with males demonstrating markedly superior engagement in physical activities, contrasted by females exhibiting a heightened propensity for mobile phone dependency. Furthermore, age and grade level manifest significant correlations with each of the four variables (*p* < 0.001). An inverse relationship is observed between physical exercise and advancements in age and academic grade, indicating a decline in physical activity as students mature and progress through the educational system. Conversely, a positive correlation is noted for self-esteem, depressive symptoms, and mobile phone dependency, suggesting that these variables tend to escalate with increasing age and grade level.

**Table 1:** The basic characteristics of demographic variables.

Variable	Type	PE	SE	DEP	MPD
Gender	Male (*n* = 1,914)	4.00 ± 0.82	2.43 ± 0.98	2.28 ± 0.87	0.68 ± 0.54
	Female (*n* = 1,872)	3.75 ± 0.82	2.49 ± 0.87	2.33 ± 0.80	0.82 ± 0.62
*T*		88.342	3.817	2.788	52.637
*p*		0.000**	0.051	0.095	0.000**
Age	12.0 (*n* = 86)	3.94 ± 0.87	2.29 ± 0.94	2.16 ± 0.87	0.64 ± 0.58
	13.0 (*n* = 1,301)	3.98 ± 0.85	2.28 ± 0.94	2.19 ± 0.85	0.70 ± 0.59
	14.0 (*n* = 1,249)	3.86 ± 0.84	2.49 ± 0.94	2.29 ± 0.82	0.76 ± 0.59
	15.0 (*n* = 1,113)	3.79 ± 0.77	2.64 ± 0.88	2.46 ± 0.82	0.81 ± 0.58
	16.0 (*n* = 37)	3.47 ± 0.76	2.64 ± 0.89	2.49 ± 0.97	0.86 ± 0.54
*F*		11.472	24.373	16.581	6.53
*p*		0.000**	0.000**	0.000**	0.000**
Grade	Grade 7 (*n* = 760)	3.96 ± 0.86	2.24 ± 0.92	2.16 ± 0.84	0.69 ± 0.59
	Grade 8 (*n* = 758)	3.88 ± 0.84	2.46 ± 0.92	2.24 ± 0.80	0.75 ± 0.59
	Grade 9 (*n* = 568)	3.78 ± 0.78	2.69 ± 0.90	2.53 ± 0.83	0.82 ± 0.57
	Grade 10 (*n* = 760)	3.88 ± 0.83	0.54 ± 0.61	0.52 ± 0.56	0.36 ± 0.58
	Grade 11 (*n* = 758)	3.81 ± 0.83	0.58 ± 0.61	0.61 ± 0.59	0.45 ± 0.63
	Grade 12 (*n* = 182)	3.64 ± 0.77	0.67 ± 0.63	0.67 ± 0.60	0.47 ± 0.62
*F*		30.258	15.864	21.231	10.306
*p*		0.000**	0.000**	0.000**	0.000**

PE, physical exercise; MPD, mobile phone dependency; SE, self-esteem; DEP, depression.

### 3.3 Correlation matrix of the variables

Table 2 illustrates that there are significant correlations among the study variables. Specifically, physical exercise is significantly and negatively correlated with mobile phone dependency (*r* = −0.283, *p* < 0.01) and depression (*r* = −0.400, *p* < 0.01), while it is significantly and positively correlated with self-esteem (*r* = 0.257, *p* < 0.01). Mobile phone dependency is significantly and positively correlated with depression (*r* = 0.540, *p* < 0.01) and significantly and negatively correlated with self-esteem (*r* = −0.129, *p* < 0.01). Depression is also significantly and negatively correlated with self-esteem (*r* = −0.396, *p* < 0.01).

**Table 2:** The mean, standard deviations, and correlation matrix of the variables.

	M	*SD*	Gender	Age	Grade	PE	MPD	DEP	SE
Age	14.384	1.6	−0.157**	1					
Grade	2.693	1.044	−0.119*	0.764**	1				
PE	4.056	1.07	0.063	0.160**	0.121*	1			
MPD	3.149	0.661	−0.091	0.100	0.153**	−0.283**	1		
DEP	3.399	0.663	−0.102	0.102	0.148**	−0.400**	0.540**	1	
SE	3.133	0.696	−0.149**	0.172**	0.199**	0.257**	−0.129**	−0.396**	1

** indicates a significant correlation at the 0.01 level (two-tailed); * indicates a significant correlation at the 0.05 level (two-tailed).

PE, physical exercise; MPD, mobile phone dependency; SE, self-esteem; DEP, depression.

### 3.4 Serial mediating effect analysis of self-esteem and depression

This study utilized the Process macro procedure in SPSS, controlled for demographic variables (gender, age, and grade), and selected a bootstrap sample size of 5,000 at a 95% confidence interval to test the serial mediating effects of self-esteem and depression.

Firstly, multiple regression analysis indicated that physical exercise has a significant negative predictive effect on mobile phone dependency (*B* = −0.173, *p* < 0.01). When physical exercise, self-esteem, and depression were included together in the regression equation, the predictive effect of physical exercise on mobile phone dependency remained significant (*B* = −0.057, *p* < 0.01). The positive predictive effect of physical exercise on self-esteem was significant (*B* = 0.108, *p* < 0.01), as was the negative predictive effect on depression (*B* = −0.291, *p* < 0.01). Self-esteem had a significant negative predictive effect on depression (*B* = −0.679, *p* < 0.01) and a significant negative predictive effect on mobile phone dependency (*B* = −0.160, *p* < 0.01). Depression had a significant positive predictive effect on mobile phone dependency (*B* = 0.365, *p* < 0.01) (Table 3).

**Table 3:** Regression model.

	M1			M2			Y			Y		
	*B*	*SE*	*t*	*B*	*SE*	*t*	*B*	*SE*	*t*	*B*	*SE*	*t*
Constant	6.509**	0.724	8.989	60.272**	1.485	40.586	43.712**	1.04	42.041	22.278**	1.771	12.58
X	0.108**	0.016	6.935	−0.291**	0.031	−9.289	−0.173**	0.022	−7.701	−0.057**	0.021	−2.659
M1				−0.679**	0.074	−9.121				−0.160**	0.051	3.157
M2										0.365**	0.025	14.762
*R* ^2^	0.066			0.252			0.08			0.307		
Adjusted *R*^2^	0.065			0.25			0.079			0.304		

The first Y column shows the direct effect of X on Y, while the second Y column shows the indirect effect of X on Y after controlling for M1 and M2.

Secondly, the mediation effect was tested. Table 4 shows that self-esteem and depression mediate the relationship between physical exercise and mobile phone dependency, with a mediation effect value of −0.116. Its 95% confidence interval is [−0.239, −0.140], which does not contain 0, indicating a significant mediation effect, accounting for 100% of the total effect of physical exercise on mobile phone dependency. The mediation effect includes three indirect effect paths: the indirect effect 1 through the “physical exercise—depression—mobile phone dependency” pathway (−0.076); the 95% confidence interval is [−0.224, −0.128], which does not contain 0, indicating a significant indirect effect of the mediating variable, accounting for 65.52% of the total effect of physical exercise on mobile phone dependency. The indirect effect 2 through the “physical exercise—self-esteem—depression—mobile phone dependency” pathway (−0.040); the 95% confidence interval is [−0.063, −0.027], which does not contain 0, indicating a significant indirect effect of the mediating variable, accounting for 34.48% of the total effect of physical exercise on mobile phone dependency. The indirect effect through the “physical exercise—self-esteem—mobile phone dependency” pathway did not reach a significant level (the confidence interval contains 0).

**Table 4:** Path coefficient test.

	Effect	Boot SE	BootLLCI	BootULCI	*z*	*p*	Effect value
X ⇒ M1 ⇒ Y	0.017	0.011	0.009	0.052	1.55	0.121	−14.65%
X ⇒ M2 ⇒ Y	−0.076	0.024	−0.224	−0.128	−4.513	0.000	65.52%
X ⇒ M1 ⇒ M2 ⇒ Y	−0.040	0.009	−0.063	−0.027	−2.919	0.004	34.48%
X= > Y	−0.116	0.025	−0.239	−0.14	−4.662	0.000	100%

**Figure 2:**
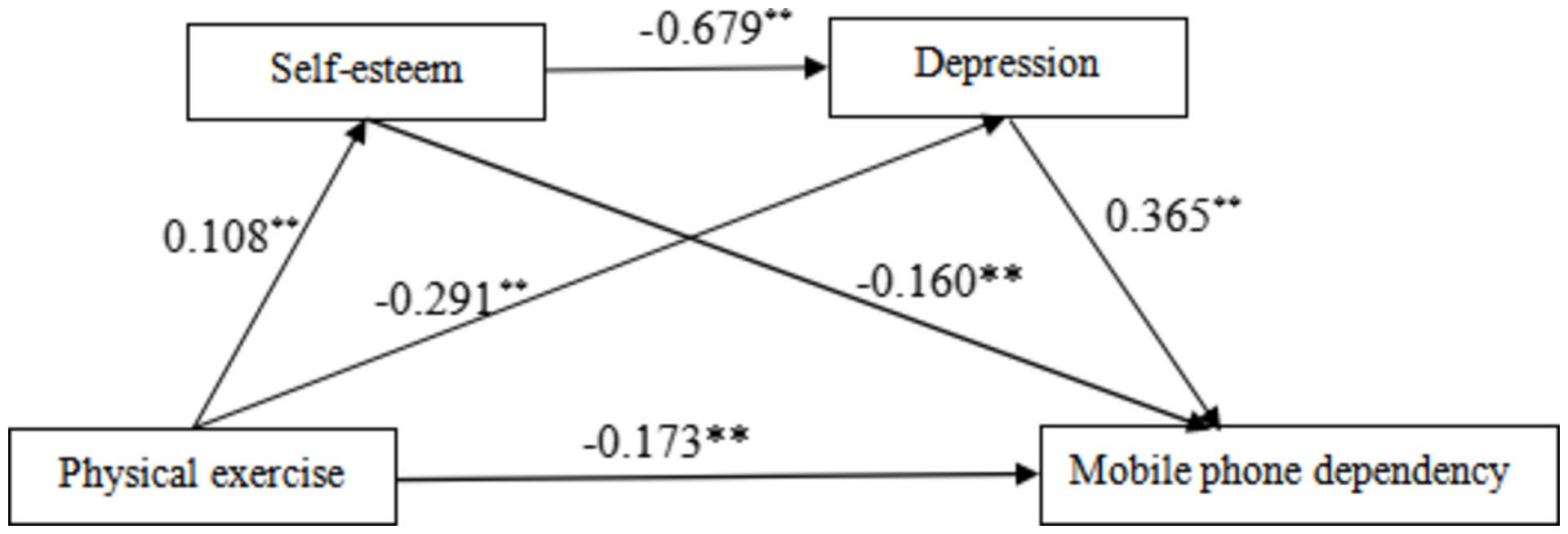
Serial mediation effect test diagram. The path coefficients in the diagram are standardized coefficients, and ** indicates *p* < 0.01.

The detailed path model of how physical exercise affects mobile phone dependency is shown in Figure 2. The results indicate that physical exercise significantly influences mobile phone dependency through the single mediation of depression and the serial mediation of self-esteem and depression, while the single mediation effect of self-esteem is not significant. This suggests that physical exercise may primarily reduce mobile phone dependency by lowering depression levels and may also indirectly reduce mobile phone dependency by enhancing self-esteem, which in turn reduces depression levels.

## 4 Discussion

This study reveals the serial mediating role of self-esteem and depression in the process of physical exercise affecting mobile phone dependency, representing an active exploration in the field of how physical exercise inhibits phone dependency. Theoretically, it fills the gaps in the mechanism of intervention for mobile phone dependency and deepens the research on how physical exercise enhances self-control and efficacy. From a practical life perspective, regular physical exercise can significantly improve an individual’s self-control ability, reduce the continuous deterioration of mobile phone dependency behavior, and save the physical and mental health of middle school students, which has practical guidance value.

### 4.1 The relationship between physical exercise and mobile phone dependency

The results of the study indicated that physical exercise can significantly negatively predict mobile phone dependency (*r* = −0.32, *p* < 0.001), which is consistent with our research hypothesis H1, that physical exercise can negatively predict mobile phone dependency behavior. This empirical result is consistent with the Self-Determination Theory, which advocates that individuals need to meet three basic psychological needs: autonomy, competence, and relatedness in the social environment, and physical exercise can fulfill these needs (Fernández-Espínola et al., 2022). Specifically, an individual’s participation in physical exercise during leisure time can achieve physical and mental pleasure and autonomous communication. In the long-term persistence, goals will be gradually achieved, indirectly obtaining more job competence, and also getting the recognition and praise of others, enhancing emotional communication between each other, and bringing satisfaction to relational needs (Riiser et al., 2014). In contrast, excessive use of mobile phones will only increase the individual’s maladaptation and emotional disorder. Although short-term inner pleasure can be satisfied, long-term indulgence will only bring psychological and pathological mental disorders, severely hindering the physical and mental health of adolescents and leading to an exacerbation of dependency symptoms (Noruzi Kuhdasht et al., 2018).

In the multiple regression analysis results, we found that after controlling for demographic variables such as gender, age, and grade, physical exercise still has a significant negative predictive effect on mobile phone dependency (β = −0.29, *p* < 0.001). This result indicates that the more middle school students participate in physical exercise during their leisure time, the lower their degree of mobile phone dependency will be. This result is consistent with the conclusions of previous scholars, such as the negative correlation between college students’ physical exercise levels and mobile phone dependency (Liu et al., 2022).

However, as researchers, we need to pay attention to the fact that there may be some non-linear relationships between physical exercise and mobile phone dependency. For example, some studies have shown that students with different frequencies and intensities of physical exercise also have significant differences in the degree of mobile phone dependency (Huo, 2021). For instance, scholars believe that moderate-intensity physical exercise has the best effect, and its dosage results show the most significant effect on reducing mobile phone dependency, while excessive physical exercise may exacerbate the use of mobile phone dependency (Yang et al., 2019). This suggests that there may be other moderating variables between the two, and future research needs further exploration and excavation to sort out the best effect dosage of physical exercise intervention.

In addition, although this study has explored the relationship between physical exercise and mobile phone dependency, this relationship and its mechanism still need further exploration. Perhaps it can be achieved through other paths such as improving self-efficacy and self-control ability, which are the main variables we need to consider.

### 4.2 The mediating role of self-esteem

The results of the study revealed that self-esteem exerts a partial mediating influence on the impact of physical exercise on mobile phone dependency, substantiating our preliminary hypothesis, H2.

Firstly, the correlation matrix analysis demonstrated a significant positive association between physical exercise and self-esteem (*r* = 0.38, *p* < 0.001), coupled with a significant negative association between self-esteem and mobile phone dependency (*r* = −0.41, *p* < 0.001). These findings align with the Self-Determination Theory, which posits that physical exercise can bolster self-esteem by fulfilling individuals’ fundamental psychological needs (Alsaeed et al., 2023; Tsartsapakis et al., 2024). Specifically, middle school students, by engaging in a variety of sports activities, can satisfy their intrinsic psychological needs for individualization and autonomy. This participation gradually garners recognition and praise from others (Chen et al., 2022), fulfilling their sense of competence and achievement. Concurrently, it enhances interpersonal relationships and emotional communication, advancing relational needs (Liu et al., 2023). The satisfaction of these basic needs significantly elevates an individual’s self-concept and social value, thereby augmenting self-esteem levels.

Secondly, employing the Bootstrap method to assess the mediating effect, the results indicated that the mediating influence of self-esteem remains significant after controlling for gender, grade, and age (indirect effect = −0.15, 95% *CI* = [−0.20, −0.10]). This suggests that physical exercise indirectly diminishes mobile phone usage by enhancing individuals’ self-esteem. In particular, regular physical exercise during leisure time can boost self-esteem, self-efficacy, and control, empowering individuals to confront life’s challenges and setbacks with greater resilience and reducing reliance on mobile phones. This is corroborated by studies showing that college students who engage in regular physical exercise during their leisure time can significantly elevate their self-esteem and negatively predict the degree of mobile phone dependency (Shang et al., 2021). A longitudinal study also discovered that physical exercise can indirectly decrease mobile phone dependency by enhancing self-esteem levels (Fasciano et al., 2021). The data further indicate that while the mediating effect of self-esteem is significant and the confidence interval does not include zero, a significant direct effect along the path remains (direct effect = −0.18, *p* < 0.001). This suggests that self-esteem’s role is one of partial mediation in the pathway from physical exercise to mobile phone dependency, hinting at the presence of additional mediating mechanisms.

Moreover, as this study is a foundational cross-sectional analysis, it circumscribes our ability to infer causality. Although numerous prior studies have validated our results, the possibility of reverse causality—where the degree of mobile phone dependency could influence behaviors such as physical exercise—cannot be overlooked. For instance, individuals with high self-esteem may be more prone to engage in physical exercise as a means of seeking social value and identity, whereas those highly dependent on mobile phones may participate minimally in physical activities. Future research should, therefore, contemplate alternative methodologies or quasi-experimental designs to elucidate and explore the interrelationships between variables.

In conclusion, the findings of this study reaffirm the pivotal role of self-esteem in the influence of physical exercise on mobile phone dependency, offering novel insights for understanding and addressing the issue of mobile phone addiction among middle school students. Moving forward, it is imperative to not only encourage youth to participate in sports activities outside the classroom but also to focus on enhancing their self-esteem through these activities, thereby effectively aiding them in overcoming mobile phone addiction.

### 4.3 The mediating role of depression

The results of the study highlighted the significant mediating role of depression in the pathway from physical exercise to mobile phone dependency, thereby validating Hypothesis 3 and offering a novel perspective for understanding the influence of physical exercise on mobile phone dependency.

Initially, the correlation matrix analysis discloses an inverse relationship between physical exercise and depression (*r* = −0.35, *p* < 0.001), concurrent with a positive correlation between depression and mobile phone dependency (*r* = 0.43, *p* < 0.001). These observations are in harmony with prior research and are congruent with the tenets of Self-Determination Theory (Alsaeed et al., 2023). Advocates of this theory suggest that physical exercise can mitigate internal malaise and emotional turbulence through psychological modulation, thus suppressing the manifestation of depressive sentiments (Liu J. et al., 2024; Liu X. et al., 2024). In detail, physical exercise presents multiple benefits in the mitigation of depressive symptoms: physiologically, it stimulates the release of endorphins and other eudaemonic hormones, thereby directly ameliorating an individual’s affective state (Pangkahila et al., 2016); psychologically, it markedly augments self-efficacy and self-regulation, fulfilling the individual’s sense of competence and fundamental psychological requisites (Xu and Tang, 2024); and from a socio-relational vantage point, physical exercise activities can enhance interpersonal communication, effectively palliate inter-personal dynamics, resolve discord, and fulfill the yearning for social interaction (Cheng et al., 2020). When these mechanisms are concurrently or partially activated, they can markedly diminish the incidence of depressive symptoms.

Subsequently, the Bootstrap method was engaged to ascertain the mediating effect. Post the control of demographic covariates, the findings further evince that the mediating effect of depression is substantive (indirect effect = −0.13, 95% *CI* = [−0.18, −0.09]). This implicates that physical exercise indirectly attenuates the intensity of mobile phone dependency by reducing the individual’s depressive affect. Specifically, a regimen of regular physical exercise can notably lower an individual’s depressive levels, emboldening them to confront life’s adversities and challenges with greater audacity, curtailing the reliance on mobile phones for emotional palliation, and consequently diminishing the reliance on mobile phones.

This discovery aligns with the conclusions of extant scholarly work. Scholars posit a positive correlation between depression and mobile phone dependency, with physical exercise posited as an alleviant of depression, thereby potentially curbing the escalation of mobile phone dependency symptoms (Du et al., 2024). This study further substantiates the mediating role of depression in the influence of physical exercise on mobile phone dependency, augmenting the empirical evidence for this mechanism. However, it is imperative to acknowledge that while the mediating effect of depression is significant and the confidence interval excludes zero, a direct effect of physical exercise on mobile phone dependency is also extant (direct effect = −0.16, *p* < 0.001), indicating that depression may only play a partial mediating role in this pathway, with other mechanisms potentially at play.

Furthermore, it is essential to contemplate the possibility of bidirectional pathways, although current evidence may not fully corroborate this. For instance, depression may predispose individuals to increased mobile phone dependency, with excessive use potentially exacerbating depressive symptoms. Thus, future research should incorporate longitudinal multi-time assessments to further elucidate the causal relationships and underlying mechanisms between these variables.

In sum, the study’s outcomes reiterate the pivotal role of depression in the process by which physical exercise influences mobile phone dependency, providing novel insights for a deeper comprehension of adolescent mobile phone dependency. It underscores the necessity to consider underlying emotional states, particularly depressive affect, when devising interventions for mobile phone dependency, advocating for a multifaceted approach to intervention strategies.

### 4.4 The serial mediating role of self-esteem and depression

Following an exploration of individual mediating roles, this study further examines the serial mediating role of self-esteem and depression. Through regression analysis and path coefficient test outcomes, it was determined that self-esteem and depression serially mediate the impact of physical exercise on mobile phone dependency, thereby confirming the H4 hypothesis of this study and offering a holistic perspective on the intricate relationship through which physical exercise affects mobile phone dependency.

Initially, the correlation analysis matrix revealed a significant negative correlation between self-esteem and depression (*r* = −0.47, *p* < 0.001), aligning with scholarly conclusions and cognitive behavioral theory (Heekerens et al., 2023). This theory suggests that individuals with lower self-esteem are more susceptible to negative emotions and attribution styles, thereby increasing their risk of depressive symptoms (Wang et al., 2023).

Subsequently, a Bootstrap method was utilized to test for serial mediating effects. After controlling for demographic variables, the serial mediating effect of self-esteem and depression was found to be significant (indirect effect = −0.08, 95% *CI* = [−0.11, −0.05]), with the confidence interval not containing zero. Specifically, the expression path of this serial mediating effect is delineated as physical exercise → self-esteem → depression → mobile phone dependency. This pathway implies that physical exercise initially elevates an individual’s self-esteem, subsequently reduces the level of depression, and ultimately culminates in diminished mobile phone dependency. This path can be dissected into three segments: the influence of physical exercise on self-esteem, where physical activity fulfills the individual’s need for subjectivity and autonomy, thereby indirectly enhancing self-esteem levels; the influence of self-esteem on depression, with higher self-esteem aiding in the formation of accurate and objective cognitive evaluations, bolstering psychological resilience, and mitigating the risk of depression; and the influence of depression on mobile phone dependency, where a reduction in depressive levels lessens the reliance on mobile phones for psychological solace and social interaction, thereby alleviating feelings of loneliness and solitude, and reducing excessive engagement with mobile devices. The uncovering of this result enriches the existing theoretical framework and provides a comprehensive analysis of the complex mechanisms by which physical exercise affects mobile phone dependency. The mechanism of action not only uncovers insights from a singular perspective but also elucidates the interplay among multiple variables, offering a more nuanced and detailed understanding of adolescent mobile phone dependency issues.

It is important to note, however, that while the serial mediating mechanism is evident, a direct effect of physical exercise on mobile phone dependency is also present (direct effect = −0.11, *p* < 0.01), with the confidence interval excluding zero. This indicates that the serial mediating mechanism of self-esteem and depression accounts for only a portion of the relationship, suggesting the presence of other variable interactions.

In conclusion, the findings of this study underscore the serial mediating mechanism of self-esteem and depression in the impact of physical exercise on mobile phone dependency, offering novel insights for a comprehensive understanding and intervention of mobile phone dependency. Future practical initiatives should encourage increased participation in physical exercises and focus on enhancing self-esteem, regulating emotional stability, and alleviating depressive emotions, thereby effectively addressing the issue of mobile phone dependency. This highlights the necessity for a multifaceted approach to intervention strategies to achieve targeted outcomes.

However, this study has several limitations. First, although a large sample size (*n* = 3,426) and multiple validated instruments were used, the use of convenience sampling may limit the generalizability of the findings. Future research could consider random sampling to improve external validity. Second, this study focused primarily on middle school students, and the results may not be directly applicable to other age groups or populations. Future studies could extend the research to different age groups or other cultural contexts to further validate the effects of physical exercise on mobile phone dependency and its mechanisms. Additionally, while the scales used in this study demonstrated good reliability and validity, self-reported data may introduce social desirability bias. Future research could incorporate experimental designs or multiple data collection methods (e.g., behavioral monitoring, parent reports) to enhance the reliability of the results.

Looking ahead, future research could further explore the influence of variables such as family support and school environment on mobile phone dependency, particularly how these factors interact with physical exercise interventions to develop effective strategies for addressing mobile phone dependency among adolescents.

## 5 Conclusion

This study investigates the mechanisms through which physical exercise affects mobile phone dependency among middle school students and arrives at the following conclusions: First, physical exercise is significantly and negatively correlated with mobile phone dependency, indicating that increased physical exercise can help reduce mobile phone dependency behaviors among students. Second, self-esteem and depression play significant mediating roles in this relationship. Self-esteem enhances individual confidence, mitigating the negative effects of mobile phone dependency, while depression, by improving emotional states, further facilitates the reduction of mobile phone dependency through physical exercise. Finally, physical exercise not only affects mobile phone dependency through the indirect effects of self-esteem and depression but also demonstrates the significant role of physical exercise in promoting students’ physical and mental health.

## Data Availability

The original contributions presented in the study are included in the article/supplementary material, further inquiries can be directed to the corresponding author.
